# High Incidence of Herpes Simplex Virus-1 in Cord Blood and Placenta Infection of Women in Southern Brazil

**DOI:** 10.1055/s-0039-1700794

**Published:** 2020-01

**Authors:** Emiliana Claro Avila, Fabiana Finger-Jardim, Carla Vitola Gonçalves, Vanusa Pousada da Hora, Marcelo Alves Soares, Ana Maria Barral de Martínez

**Affiliations:** 1 Molecular Biology Laboratory, School of Medicine, Universidade Federal do Rio Grande, Rio Grande, Brazil; 2 Center for Obstetrics and Gynecology, School of Medicine, Universidade Federal do Rio Grande, Rio Grande, Rio Grande do Sul, Brazil; 3 Oncovirology Program, Instituto Nacional do Câncer (INCA), Rio de Janeiro, Rio de Janeiro, Brazil

**Keywords:** HSV-1, vertical transmission, placenta, umbilical cord, herpesvirus, HSV-1, transmissão vertical, placenta, cordão umbilical, herpesvírus

## Abstract

**Objective** Estimate the prevalence of human herpesvirus type 1 HSV-1 DNA in placental samples, its incidence in umbilical cord blood of newborns and the associated risk factors.

**Methods** Placental biopsies and umbilical cord blood were analyzed, totaling 480 samples, from asymptomatic parturients and their newborns at a University Hospital. Nested polymerase chain reaction (PCR) and gene sequencing were used to identify the virus; odds ratio (OR) and relative risk (RR) were performed to compare risk factors associated with this condition.

**Results** The prevalence of HSV-1 DNA in placental samples was 37.5%, and the incidence in cord blood was 27.5%. Hematogenous transplacental route was identified in 61.4% from HSV-1^+^ samples of umbilical cord blood paired with the placental tissue. No evidence of the virus was observed in the remaining 38.6% of placental tissues, suggesting an ascendant infection from the genital tract, without replication in the placental tissue, resulting in intra-amniotic infection and vertical transmission, seen by the virus in the cord blood. The lack of condom use increased the risk of finding HSV-1 in the placenta and umbilical cord blood.

**Conclusion** The occurrence of HSV-1 DNA in the placenta and in cord blood found suggests vertical transmission from asymptomatic pregnant women to the fetus.

## Resumo

**Objetivo** Estimar a prevalência do DNA do vírus herpes humano 1 (HSV-1) em amostras de placenta, sua incidência no sangue do cordão umbilical de recém-nascidos e fatores de risco associados.

**Métodos** Biópsias de placenta e de sangue de cordão umbilical foram analisadas, totalizando 480 amostras de parturientes assintomáticas e seus recém-nascidos em um hospital universitário. Reação de cadeia de polimerase (RCP) *nested* e sequenciamento gênico foram usados para identificar o vírus; odds ratio (OR) e risco relativo (RR) foram realizados para comparar os fatores de risco associados à essa condição.

**Resultados** A prevalência do DNA do HSV-1 em amostras de placenta foi de 37,5%, e a incidência no sangue do cordão foi de 27,5%. A via transplacentária hematogênica foi identificada em 61,4% das amostras de HSV-1 + do sangue do cordão umbilical, pareadas com o tecido placentário. Nenhuma evidência do vírus foi observada nos restantes 38,6% dos tecidos placentários, sugerindo uma infecção ascendente do trato genital. A falta de uso do preservativo aumentou o risco de encontrar o HSV-1 na placenta e no sangue do cordão umbilical.

**Conclusão** A ocorrência de DNA do HSV-1 na placenta e no sangue do cordão umbilical sugere uma transmissão vertical de gestantes assintomáticas para o feto.

## Introduction

Human herpesvirus type 1 (HSV-1) is a ubiquitous neurotropic virus in humans. The main characteristics are the lifelong latent/persistent infection in the sensory ganglia innervating the primary infection site, and the production of vesicular lesions upon reactivation.^1^ ^2^ Typically associated with orofacial lesions, HSV-1 has emerged as a pathogen of genital infections, especially in the Americas among people between 15 and 49 years old, which is the reproductive age group.^2^ ^3^ ^4^

Genital HSV-1 is the main cause of the first episode of genital herpes in women in high income countries, as its seroprevalence is declining during childhood as a cause of oral lesions.^5^ ^6^ Consequently, adolescents and young adults have their first exposure to the virus with the initiation of sexual activity.^1^ ^7^ All over the world, ∼ 132 million women have incident or prevalent HSV infection during pregnancy.^8^ The first estimate of global neonatal herpes infection incidence predicts that the Americas have the highest regional rate due to genital HSV-1 infection.^7^ Overall seroprevalence of antibodies against HSV-1 of 67.2% was identified among young people during a study managed in Brazil.^9^ Another study investigated the prevalence of HSV-1 and HSV-2 by polymerase chain reaction (PCR) in cervical samples of 261 Brazilian women and found the occurrence of HSV-1 in 23% of the samples, while 5.4% had the HSV-2 DNA detected.^3^

During the asymptomatic virus shedding, the virus can be transmitted to the partner or even to the newborn during labor.^10^ ^11^ ^12^ The infant can become infected during pregnancy, labor or in the postnatal period.^12^ Congenital infections, not resulting in miscarriage, may affect the infant in several ways, including skin or eye lesions (cataracts, chorioretinitis or microphthalmia), neurological calcifications, microcephaly, seizures, delayed growth, and psychomotor developmental problems.^11^ ^13^

The present study aimed to simultaneously investigate the incidence of HSV-1 in neonatal cord blood and the prevalence of HSV-1 DNA in placental tissue of parturient women by correlating risk factors associated with infection and vertical transmission.

## Methods

The present work was carried out as an observational study designed to evaluate the prevalence of HSV-1 in placental samples of parturient women and the incidence of HSV-1 in cord blood samples from their newborns. Specimens were collected between March 2011 and March 2014, using a convenience sampling strategy. All of the parturients who agreed to participate voluntarily by a signed informed consent were included in the study. Patients < 18 years old were allowed to participate by the consent of the legal guardian. Patients with mental disabilities or unable to express their wishes were automatically excluded from the study. The sample size was calculated on the basis of a presumed 3.3–28.0% HSV-1 prevalence in the placenta, with associated 95% confidence intervals (CIs) using Epi-Info 7.0 (Centers for Disease Control and Prevention, Atlanta, GA, USA).^14^ ^15^ ^16^ ^17^ ^18^ The sample consisted of 160 women under medical care at the Obstetric ward from the Hospital Universitário Dr. Miguel Riet Correa Jr. (HU/UFRG, in the Portuguese acronym), a University hospital in Rio Grande, southern Brazil. Clinical examination was performed in all women when they were admitted to the obstetric ward.

Collection of umbilical cord blood samples and placental tissue biopsies were performed as previously described by Finger-Jardim et al.^19^ Subsequently, placenta samples were stored in TE buffer (10mM Tris-HCl pH 8.0; 1mM EDTA) at - 20°C, and the umbilical cord blood at - 4° C until further processing.

DNA extraction from umbilical cord blood was performed with the PureLink Genomic DNA Mini kit (Invitrogen - Life Technologies, Carlsbad, CA, USA), according to the specifications of the manufacturer. DNA extraction from placental tissue was performed using an adapted protocol of the mentioned commercial kit as previously described.^18^ DNA samples were stored at - 20°C until used, and its quality was assessed by amplification of the human *CCR2* gene. Polymerase chain reaction products were visualized by UV light after electrophoresis on 1.5% agarose gels stained with Blue Green Loading Dye (LGC Biotecnologia, São Paulo, SP, Brazil).

Detection of HSV-1 in placental tissue and blood samples was determined by nested PCR using an adapted version of specific protocols to detect the virus.^18^ ^20^ ^21^ ^22^ The two consecutive PCR reactions used 5uL of the DNA template in the first round and 1.5 uL product from the first round in the second round, respectively. The reagents used in the reaction were: 1X PCR buffer, 2 mM MgCl2, 0.5 mM dNTPs, 1U Platinum Taq DNA polymerase enzyme (Life Technologies, Carlsbad, CA, USA), Milli-Q H_2_O q.s.p. and HSV-1. Previously described primers by Aurelius et al^20^ were employed to amplify a fragment of 138pb of the HSV-1 *gD* gene. Samples were processed with positive and negative controls in each reaction, and with a blank reaction (no DNA added). The positive control was obtained from a dead cell suspension containing the virus (Vero cell DNA, simian DNA virus-positive, Virology Laboratory of the Universidade Federal do Rio Grande do Sul). Polymerase chain reaction products were subjected to electrophoresis on 2% agarose gels, stained with Blue Green and visualized by UV illumination in an LPIX Transilluminator (Loccus, São Paulo, Brazil). Positive samples were repeated at a new reaction with positive and negative controls and a blank reaction. The positive samples were purified with Illustra GFX PCR DNA and Gel Band Purification Kit (GE Healthcare Life Sciences, Piscataway, NJ, USA) and sequencing was performed using an ABI Prism BigDye Terminator Cycle Sequencing Ready Reaction Kit (Applied Biosystems, Foster City, CA, USA) in an automated ABI 3130XL analyzer (Thermo Scientific, Waltham, MA, USA). The sequences found in this study were compared to HSV-1 sequences available in the GenBank database, using the BLASTn algorithm.

Data on risk factors for HSV-1 infection were obtained by a self-reported questionnaire and hospital database. Clinical, gynecological, laboratory and sociodemographic variables were evaluated for each participant. An active search was performed on the charts of the neonates presenting positive HSV-1 by PCR from umbilical cord blood samples. The Chi-squared test was used to compare categorical variables: age, educational attainment, skin color, marital status, income, age at onset of sexual intercourse, number of lifetime partners, contraception method, comorbid STDs (sexual transmitted diseases), number of gravidity, mode of delivery, history of abortion, time between rupture of membranes and delivery, and gestational time. The OR for each variable was calculated, potential risk factors and protective factors were investigated, and frequency distributions and percentages were determined. Differences were considered statistically significant when *p* < 0.05. Multivariate analysis with Poisson regression was also performed, followed by the construction of a hierarchical linear model, which incorporated variables with *p* ≤ 0.20 in the crude analysis. The first level consisted of demographic and socioeconomic variables, while in the second included the variables comprising risk factors for HSV-1 infection. All analyzes were performed using SPSS Statistics for Windows, Version 12.0 (SPSS Inc., Chicago, IL, USA) and Epi Info v.7.0.

The present study was approved by the Research Ethics Committee at the Health Area (CEPAS, in the Portuguese acronym) of the University Federal do Rio Grande (UFRG, in the Portuguese acronym) (CEPAS N° 54/2011). All of the participants (or their legal guardians, when appropriate) provided written informed consent for participating in the study

## Results

Along the whole study, 480 specimens were analyzed, comprising 160 placentas (maternal and fetal sides = 320 samples) and 160 newborns cord blood samples. All of the samples were tested. The prevalence of HSV-1 found in the placenta was 37.5% (*n* = 60) (maternal, fetal, or both interfaces infected, showing tissue permissiveness to the virus) and the incidence in cord blood was 27.5% (*n* = 44). Vertical hematogenous transplacental transmission was identified in 27 (61.4%) umbilical cord blood samples. HSV-1 was present in cord blood, without evidence of virus in the 17 (38.6%) corresponding placentas, suggesting intra-amniotic infection without placental involvement (Table 1).

**Table 1:** Sociodemographic, Obstetrical and Gynecological Profile of Parturient Women, Stratified by HSV-1 Positivity in the Sample

Variable/ Category (*n*)	*n* (%)*	HSV-1+ placenta *n* (%)*	Prevalence Ratio	95%CI	*p-value;* c b	HSV-1+ cord blood n (%)*	Odds Ratio	95% CI	*p-value* b
Age (years old) (133)									
≤20	35 (26.3)	14 (40)	1.0		0.39	7 (20)	1.0		*0.22*
21-30	71 (53.4)	34 (47.9)	1.19	0.74–1.92		25 (35.2)	2.17	0.83–5.68	
31-40	27 (20.3)	9 (33.3)	0.83	0.42–1.62		10 (37)	2.35	0.75–7.34	
Educational attainment (years) (150)									
≥9	83 (55.3)	30 (36.1)	1.0		*0.81*	23 (27.7)	1.0		*0.45*
≤8	67 (44.7)	23 (34.3)	0.94	0.61–1.47		15 (22.4)	0.75	0.35–1.59	
Skin color (self-reported) (155)									
White	99 (63.9)	38 (38.4)	1.0		*0.58*	29 (29.3)	1.0		*0.41*
Non-white	56 (36.1)	19 (33.9)	1.13	0.72–1.76		13 (23.2)	0.72	0.34–1.55	
Marital Status (155)									
Stable Partner	117 (75.5)	43 (36.8)	1.0		*0.74*	35 (29.9)	1.0		*0.16*
No Stable Partner	38 (24.5)	14 (37.8)	1.0	0.62–1.61		7 (18.9)	1.89	0.76–4.69	
Income (in minimum wages)^a^ (146)									
≥2	50 (34.2)	20 (40)	1.0		*0.42*	14 (28)	1.0		*0.59*
≤1	96 (65.8)	32 (33.3)	0.83	0.53–1.29		23 (24)	0.81	0.37–1.75	
Age at onset of sexual intercourse (years) (128)									
≥16	63 (49.2)	28 (44.4)	1.0		*0.49*	23 (36.5)	1.0		*0.09*
≤15	65 (50.8)	25 (38.5)	1.15	0.76–1.74		15 (23.1)	1.91	0.88–4.14	
Number of lifetime partners (124)									
1	39 (31.5)	18 (46.2)	1.0		*0.53*	15 (38.5)	1.0		*0.27*
2– 4	56 (45.1)	20 (35.7)	0.77	0.47–1.26		16 (28.6)	0.64	0.26–1.52	
≥ 5	29 (23.4)	13 (44.8)	0.97	0.57–1.64		6 (20.7)	0.41	0.13–1.26	
Contraception (130)									
Condom and/or other methods	39 (30)	13 (33.3)	1.0			6 (15.4)	1.0		
Contraception (excluding condom)	91 (70)	42 (46.2)	1.38	0.84–2.27	*0.17*	35 (38.5)	3.43	1.30–9.04	***0.009***
Comorbid STDs (153)									
No	143 (93.5)	51 (35.9)	1.0			37 (26.1)	1.0		*0.77*
Yes	10 (6.5)	4 (40)	1.11	0.50–2.45	*0.72*	3 (30)	1.22	0.30–4.99	
Gravidity (129)									
1^st^ pregnancy	53 (41.1)	21 (39.6)	1.0		*0.46*	14 (26.4)	1.0		*0.34*
≥ 2	76 (58.9)	35 (46.1)	1.16	0.76–1.75		26 (34.2)	1.44	0.66–3.13	
Mode of Delivery (159)									
Cesarean	86 (54.1)	28 (32.6)	1.0		*0.19*	28 (32.6)	1.0		*0.13*
Vaginal	73 (45.9)	31 (42.5)	1.30	0.87–1.95		16 (21.9)	0.58	0.28–1.18	
History of abortion (148)									
No	121 (81.8)	44 (36.4)	1.0		*0.43*	32 (26.4)	1.0		*0.73*
Yes	27 (18.2)	12 (44.4)	1.22	0.75–1.98		8 (29.6)	1.17	0.46–2.93	
Time between rupture of membranes and delivery (minutes) (122)									
≤360 minutes	82 (67.2)	31 (37.8)	1.0		*0.39*	21 (25.6)	1.0		*0.94*
≥360 minutes	40 (32.8)	12 (30)	0.79	0.45–1.37		10 (25)	0.96	0.40–2.31	
Gestational time (weeks)^c^ (143)									
≥38 (full-term)	117 (81.8)	51 (43.6)	1.0		*0.40*	35 (29.9)	1.0		*0.63*
≤37 (preterm)	26 (18.2)	9 (34.6)	1.79	0.45–1.40		9 (34.6)	1.24	0.50–3.04	

Abbreviations: CI, confidence interval; HSV1, human herpesvirus type 1.

* All respondents.

a Calculed as equivalence to the Brazilian minimum wage at the time of the study (approximately US$ 350.00);

b Chi-squared test.

c Calculated by means of the Capurro method; statistically significant result is indicated in bold.

Regarding the gynecological history, the only variable significantly associated with HSV-1 infection in cord blood was the use of hormonal contraception. Women who used hormonal contraception or othercontraceptive method, except for condoms, had almost 4 times more chances to present the virus in the umbilical cord blood of their neonates than those associated with condom use or another method (95%CI: 1.30–9.04; *p*  = 0.009; Table 1). There were no significant associations between the presence of HSV-1 and obstetric variables.

The presence of placental HSV-1 increased the chance of this viral infection in the umbilical cord blood (95%CI: 1.92–8.27; *p* < 0.001; OR = 3.99). Also, the presence of HSV-1 in the cord blood increased the chance of placental infection (95%CI: 1.48–3.124.43; *p*  < 0.001; RR = 2.15).

A total of 15 subjects (34%) were born with alterations, such as ocular inflammation and pustules in the genital region after vaginal delivery, limb bruising, decreased reflexes, hypotonia and thrombocytopenia. There was one case of hydrocephaly and one case of congenital syphilis. None had herpes diagnosis or were investigated for this infection at birth, and the mothers did not return after an active search for pediatrics care. Clinical findings such as low birthweight (data not shown) and prematurity had no significant association with cord blood incidence.

## Discussion

Vertical transmission is the passage of a pathogen from mother to child that can occur still in the uterus (hematogenous transplacental), peripartum or during the postnatal period.^23^

In the present study, the prevalence of HSV-1 DNA found in placenta samples was 37.5% (*n* = 60), which is considered high when compared with other studies that also identified the occurrence of HSV only in the placenta and reported prevalence rates between 2.6 and 28%.^14^ ^15^ ^16^ ^17^ ^18^ These findings demonstrate that the virus prevalence in placental tissue is frequent among the pregnant women who joined the study. Therefore, the monitoring and tracking of the virus during pregnancy is quite relevant, considering the possible neonatal complications that might occur. However, it is important to note that most studies have investigated placental tissue from unsuccessful pregnancies and, differently from what was analyzed in the present study, few of them investigated the difference between HSV-1 and HSV-2.^15^ ^17^ Finger-Jardim et al^18^ found a 29.9% prevalence of HSV-1 in the fetal side of the placental tissue, using the same methodology applied in the present work, and women who reported no use of condom during sexual intercourse had two times more chances to be HSV-1-positive in the placental tissue. Therefore, the placental tissue seems to play an important role as a reservoir for the virus. Burgos et al^24^ simulated HSV-1 vertical transmission in mice and investigated the presence of viral DNA in fetuses, newborns and adult models in the offspring of mothers with latent infection. HSV-1 DNA was identified and quantified by PCR, and all subjects presented detectable levels of the virus in the central nervous system (CNS), in the placenta, and in the blood. That study demonstrated that viral DNA in the blood is common, and it reaches the placental tissue through the maternal bloodstream, clearly confirming vertical transmission of the virus in animal models.^24^ Similarly, our study presented the hematogenous transplacental route as an important pathway for HSV-1 transmission.

The present work reported an incidence of 27.5% (*n* = 44) for HSV-1 DNA in cord blood and, among these, 61.4% (*n* = 27) had the viral DNA also detected in the placenta, indicating vertical transmission from placenta through the blood. Tavakoli et al^25^ investigated the incidence of HSV DNA in newborn umbilical cord blood and found 2% for HSV-1 and 4% for HSV-2. Thirty percent of them were born by cesarean section and mothers were asymptomatic seropositive for the virus.^25^ During pregnancy, pathogens can be transmitted to the placenta by ascending infection from decidual cells (maternal side of the placenta) to the villi (fetal side).^16^

Regarding genital HSV lesions, all of the parturients were asymptomatic during clinical examination and denied genital herpetic lesions. HSV-1 induces less recurrent infections in the genital tract when compared with HSV-2, but apparently infects the newborn more easily later. However, ∼ 70% of the infected newborns have normal development if they are not affected by the disseminated form, which can be lethal.^10^ In the present study, 38.6% from the positive cord blood samples presented no evidence of virus in the respective placentas and no genital lesions, suggesting an ascending origin of the virus. In this scenario, we consider that pregnant women with asymptomatic viral shedding can transmit the virus to the newborn, as previously reported in other studies.^10^ ^11^ ^18^

The presence of the virus in the blood nourishing the fetus during pregnancy is facilitated by its occurrence in the placenta, which is a tissue shown to be permissive to HSV infection *in vitro*.^23^ ^26^ There are few studies investigating HSV vertical transmission using the cord blood as a target tissue. Finger-Jardim et al^19^ studied the prevalence of HSV-2 in the placenta, and found viral DNA in 9% of the analyzed tissues and a 1.1% incidence in the umbilical cord blood of newborns. Considering the mentioned study, membrane rupture time was significantly associated with infection, unlike the observed for HSV-1 in the present work. Another report using the same methodology applied in the present study found 28% and 29.9% of prevalence of HSV-1 DNA in maternal and fetal placental samples, and lack of condom use and vaginal delivery were identified as independent risk factors for HSV-1 infection in the maternal side of the placenta.^18^ Lack of condom use is a risk factor in the present study, increasing vertical transmission by almost four times, thus reassuring HSV-1 as a sexually transmitted infection (STI) agent and supporting the idea of ascendant infection transmission, and this association has been previously observed in other studies.^7^ ^12^ ^18^

HSV is an STI, and the use of condom during sexual intercourse is a protective method. In the present study, the use of methods for contraception (oral or injectable) lacking the physical barrier method such as condoms, was a significant variable related to detection of HSV-1 DNA in the umbilical cord blood of newborns. However, among the positive umbilical blood samples for viral DNA, almost 40% were viral-negative in the placentas, suggesting an asymptomatic ascending genital tract infection. This result showed that women who were exposed to the virus without using condoms had a risk almost four times higher of presenting viral DNA in the umbilical cord blood of their newborns. These findings support previous studies that project HSV-1 as an emergent STI.^3^ ^4^

The high incidence of HSV-1 DNA found in the umbilical cord blood of newborns suggests vertical transmission in the uterus. An active search was performed on the charts of newborns presenting HSV-1 in the umbilical cord blood, and 13.6% (*n* = 6) of them were born from mothers who underwent preeclampsia protocol. No significant association with low birthweight, prematurity and cord blood HSV-1 incidence was observed. Alterations such as vaginal postpartum ocular inflammation, limb bruising, decreased reflexes, hypotonia and pustules were reported in the medical records, but no clinic or laboratorial investigation for HSV was realized. Neonatal herpes infection can cause newborn skin and eye lesions, and these symptoms can be caused by the HSV-1 virus.^13^ ^27^ Identification of HSV-exposed infants allows resources to be focused on those at highest risk, but it is hard to identify the disease in asymptomatic or HSV-1 recurrent mothers.

Some limitations should be considered when interpreting the results of the present work. The present study was conducted in pregnant women from a single university hospital (medium-sized hospital, 185 beds) who serves only patients depending on the Brazilian public health system. Another limitation is related to the self-administered questionnaire, since patients sometimes did not answer to all questions. Also concerning this matter, there were no questions about oral injuries caused by herpes, information that would provide more rich details for the obtained results.

## Conclusion

In summary, the present study described a high prevalence of HSV-1 in placenta tissue samples, as well as a resulting high incidence in cord blood. The presence of HSV-1 in the placenta represents a risk for vertical transmission. This observation, in association with the investigated risk factors, shows the importance of virus transmission and the possibility of asymptomatic ascending infection of the genital tract. The occurrence of this virus was high in the group of women studied and, consequently, in the newborns, with these born with signs suggesting viral infection. This research contributes to better understand HSV-1 as a major infection, also being regarded as an STI that can lead to diseases in newborns. The high occurrence of vertical transmission deserves greater attention, since this virus can cause serious complications to newborns.
